# O5.5. SLEEP IN MAJOR PSYCHIATRIC DISORDERS: RESULTS FROM NATIONWIDE SUPER FINLAND STUDY

**DOI:** 10.1093/schbul/sby015.219

**Published:** 2018-04-01

**Authors:** Minna Torniainen-Holm, Erik Cederlöf, Willehard Haaki, Jarmo Hietala, Katja Häkkinen, Erkki Isometsä, Kaisla Joutsenniemi, Tuomas Jukuri, Risto Kajanne, Olli Kampman, Tuula Kieseppä, Nina Lindberg, Markku Lähteenvuo, Jouko Lönnqvist, Teemu Männynsalo, Jussi Niemi-Pynttäri, Kimmo Suokas, Jaana Suvisaari, Jari Tiihonen, Annamari Tuulio-Henriksson, Asko Wegelius, Juha Veijola, Aarno Palotie, Tiina Paunio

**Affiliations:** 1 National Institute for Health and Welfare, University of Helsinki; 2 University of Turku; 3 Institute for Molecular Medicine Finland, Niuvanniemi Hospital; 4 Helsinki University Hospital, University of Helsinki; 5 University of Oulu; 6 University of Tampere, Seinäjoki Hospital District; 7 Institute for Molecular Medicine Finland, University of Tampere; 8 City of Helsinki, Social Services and Health Care Sector; 9 University of Tampere; 10 Niuvanniemi Hospital, University of Eastern Finland, Karolinska Institutet; 11 University of Helsinki, The Social Insurance Institution of Finland; 12 Helsinki University Hospital

## Abstract

**Background:**

People with psychotic disorders demonstrate a wide spectrum of sleep abnormalities. Abnormalities include changes in total sleep time, increased sleep onset latency, increased wake-up time after sleep onset and abnormalities in sleep architecture. The study aimed to characterize the sleep difficulties in a large sample of persons with psychotic disorders and to examine association with age and gender.

**Methods:**

Altogether, 5046 persons with a major psychiatric disorder (schizophrenia: 2972, schizoaffective disorder: 640, bipolar disorder: 1097 and psychotic depression: 330) and aged 18–80 participated in a nationwide Super project. The Finnish SUPER (Finnish acronym for “Finnish study on genetic mechanisms of psychotic disorders”) study is a part of the international Stanley Global Neuropsychiatric Genomics Initiative. The results were compared with a representative general population sample of 8018 adults (Health 2000). Sleep was assessed in a self-report questionnaire. In the present study we used total sleep time, tiredness (defined as feeling more tired than other people of the same age during day time at least weakly, yes/no), difficulties in getting sleep without sleep medication often or almost daily (yes/no) and early morning or night awakenings occurring either often or nearly every night (yes/no).

**Results:**

Long sleep (> 10h) was most common in persons with schizophrenia or schizoaffective disorder reported by approximately 30% when age was 18–40. The corresponding proportion was approximately 15% in persons with bipolar disorder or psychotic depression and less than 1% in the general population. Tiredness, difficulties in getting sleep and early morning or night awakenings were reported most by persons with bipolar disorder and psychotic depression, but also persons with schizophrenia reported those more than the general population in people with under 60 years of age. Schizoaffective disorder was between schizophrenia and affective psychoses in the sleep variables. In persons over 60, the difference between the groups was smaller than in persons under 60 years of age, because sleeping long and tiredness decreased in all patient groups, and difficulties getting sleep and awakenings increased in the general population sample more than in psychosis patients.

**Discussion:**

Sleep disorders seem to be prominent in persons with major psychiatric disorders. Tiredness was common in all diagnosis groups. Long sleep was most common in schizophrenia and difficulties in getting sleep and early morning or night awakenings in affective psychoses. More research is needed on possibilities to prevent and treat sleep disorders in major psychiatric disorders.

